# Cross-software comparison shows strong agreement for quantitative indocyanine green fluorescence angiography in reconstructive surgery

**DOI:** 10.3389/fsurg.2026.1776745

**Published:** 2026-06-24

**Authors:** Guy Oster, Lasse W. P. van ‘t Hof, Daniel M. de Bruin, Caroline Driessen

**Affiliations:** 1 Department of Plastic, Reconstructive and Hand Surgery, Amsterdam UMC, Amsterdam, Netherlands; 2 Amsterdam Movement Sciences, Musculoskeletal Health, Amsterdam, Netherlands; 3 Cancer Center Amsterdam, Imaging and Biomarkers, Amsterdam, Netherlands; 4 Biomedical Engineering and Physics, Amsterdam UMC Location University of Amsterdam, Amsterdam, Netherlands

**Keywords:** flap surgery, fluorescence angiography (FA), fluorescence time curves (FTC), indocyanine green (ICG), perfusion assessment, quantitative perfusion parameters

## Abstract

**Introduction:**

Quantitative indocyanine green fluorescence angiography (Q-ICG-FA) has emerged as a promising tool for objective intraoperative perfusion assessment, yet there remains uncertainty regarding its reproducibility across different software platforms. This study aimed to evaluate the agreement of key Q-ICG-FA parameters across two independent software platforms using identical ICG-FA recordings.

**Methods:**

Eighty ICG-FA recordings from reconstructive procedures were analyzed using two software platforms: AMS and EPA. Both programs generated fluorescence-time curves (FTCs) and calculated seven perfusion parameters. The primary outcome was time-to-peak (TTP). Secondary outcomes included T_0_, F_max_, absolute mean slope inflow, normalized mean slope inflow, normalized maximum slope inflow, and normalized maximum slope outflow. Agreement between platforms was evaluated using intraclass correlation coefficients (ICC), non-parametric testing, and Bland–Altman analysis.

**Results:**

Excellent agreement was observed for TTP (ICC = 0.979, 95% CI: 0.967–0.987) and normalized mean slope inflow (ICC = 0.944, 95% CI: 0.913–0.964). Good to excellent agreement was found for T_0_, F_max_, and absolute mean slope inflow. In contrast, parameters based on normalized maximum slopes showed poor to moderate agreement, with ICC values of 0.412 for inflow and 0.315 for outflow. The Wilcoxon signed-rank test revealed significant systematic differences for six out of seven parameters, with AMS showing overall higher TTP values compared to EPA (*p* < 0.001). No significant difference was found for normalized mean slope inflow (*p* = 0.158). Bland–Altman analysis showed that while TTP values had a mean difference of +4.2 s, the limits of agreement were wide, ranging from −42.2 to +50.5 s. For the secondary outcomes, normalized mean slope inflow demonstrated the least variability and the best agreement across platforms.

**Conclusion:**

These results suggest that TTP and normalized mean slope inflow may be reliable candidates for defining quantitative perfusion thresholds, although further clinical validation is needed.

## 1 Introduction

Perfusion-related complications are a major cause of morbidity and flap loss in reconstructive surgery (1). Traditionally, surgeons assess perfusion using clinical indicators such as flap color, capillary refill, surface temperature, and wound edge bleeding. Previous studies have demonstrated that indocyanine green fluorescence angiography (ICG-FA) offers greater accuracy in perfusion assessment compared with traditional clinical evaluation (2–4). This technique allows for real-time visualization of tissue perfusion, with well-perfused regions appearing bright and poorly perfused areas appearing dark (5).

Currently, the interpretation of ICG-FA is primarily qualitative, relying on the surgeon's visual interpretation of fluorescence intensity. While clinically useful, this approach is inherently subjective and prone to interobserver variability (6). Efforts have been made to move beyond this qualitative interpretation by quantifying ICG-FA (Q-ICG-FA), with the aim of making perfusion assessment more objective and improve measurement reliability and reproducibility (7). These quantitative methods typically generate fluorescence–time curves (FTCs), from which parameters such as time-to-peak (TTP), maximum fluorescence intensity (F_max_), and slope-related measures can be derived (8, 9). Early studies suggest that these parameters may correlate with clinical outcomes and could provide a foundation for defining objective thresholds to identify tissue at risk of perfusion-related complications (10, 11).

Despite these advancements, there remains substantial heterogeneity in imaging protocols, camera systems, and analysis methods (12, 13). This may affect the intended improvements that Q-ICG-FA was designed to achieve. Moreover, it remains unclear whether different software platforms produce consistent quantitative values when analyzing the same ICG-FA data. This lack of cross-platform validation raises uncertainty about the comparability of quantitative outputs across systems. As a result, perfusion thresholds established on one platform may not be directly applied to others, limiting their clinical applicability.

To address this gap, the present study compared two Q-ICG-FA platforms using identical ICG-FA recordings. The aim was to determine the reproducibility of key perfusion parameters across systems and identify which parameters demonstrate the highest consistency. Establishing robust and comparable metrics is an essential step toward standardizing Q-ICG-FA and developing reliable perfusion thresholds.

## 2 Materials and methods

### 2.1 Study design and setting

This study is part of an ongoing prospective cohort study on ICG in reconstructive surgery (*ClinicalTrials.gov ID: NCT06129669)*. We performed a retrospective comparative analysis in a collaboration between Amsterdam UMC and an industry partner, both based in the Netherlands. The study was reviewed by the local non-WMO committee and approved by the Medical Ethics Review Committee (METC) of Amsterdam UMC (NL2025.0625).

ICG-FA recordings were obtained from an ongoing prospective cohort of patients undergoing reconstructive surgery at Amsterdam UMC. Ethical approval for the prospective cohort was obtained from the Medical Ethics Review Committee (METC) of Amsterdam UMC (NL74852.028.21). For the present analysis, the first 80 consecutive intraoperative recordings from this cohort were included, regardless of flap type or image quality.

### 2.2 Intraoperative ICG-FA protocol

During surgery, indocyanine green (IC-Green™, Akorn Pharmaceuticals, Lake Forest, IL, USA) was administered intravenously at a dose of 0.1 mg/kg, followed by a 10 mL saline flush. Near-infrared fluorescence imaging was performed using the IR-800 function of the Zeiss Tivato 700 surgical microscope (Carl Zeiss Meditec AG, Oberkochen, Germany). All recordings were obtained according to the following imaging protocol:

- Position the camera perpendicular to the imaging subject.
- Maintain a fixed working distance of 50–60 cm.
- Capture a white light image of the subject prior to ICG injection.
- Dim ambient light in the operating room during acquisition.
- Administer ICG intravenously at a dose of 0.1 mg/kg.
- Record for a duration of 5 min after injection with the following settings:
  - Focus: use *autofocus* mode.
  - Light intensity: set to 100%.
  - Gain: set to 100%.
  - Magnification settings: set to 1.0–1.6x.

### 2.3 Quantitative analysis

All intraoperative recordings were retrospectively analyzed using two Q-ICG-FA software programs: AMS, an in-house platform developed at Amsterdam UMC, and an external, non-commercial post-processing algorithm. The latter is still under development and therefore anonymized. We further refer to this algorithm as External Processing Algorithm (EPA).

For each recording, a region of interest (ROI) was first defined in the AMS software as a circular area (5–10 mm in diameter) positioned in the center of the image. To ensure identical analysis between platforms, ROI coordinates were exported from AMS as CSV files and converted into the JSON format required for EPA using a custom-built converter tool developed at Amsterdam UMC. This approach was chosen to ensure that both platforms analyzed identical ROIs, thereby minimizing ROI-related variability and allowing potential inter-platform differences to be attributed primarily to software-specific signal processing.

To reduce signal noise and ensure a stable FTC, both AMS and EPA applied digital filtering to the raw fluorescence data prior to parameter calculation. In AMS, the FTC is processed using a fourth-order low-pass Butterworth filter with a cutoff frequency of 0.1 Hz. This approach smooths high-frequency fluctuations while preserving the overall curve shape. All quantitative parameters are derived from the resulting smoothed curve.

EPA offers users a choice between two smoothing methods: the Whittaker-Henderson filter and a moving average filter, with the latter being set as the default. When using the moving average method, the degree of smoothing is controlled by the filter width, which adjusts the size of the averaging window. In this study, the moving average filter with a width of 77 was used, which was the default in this software.

**Figure 1:**
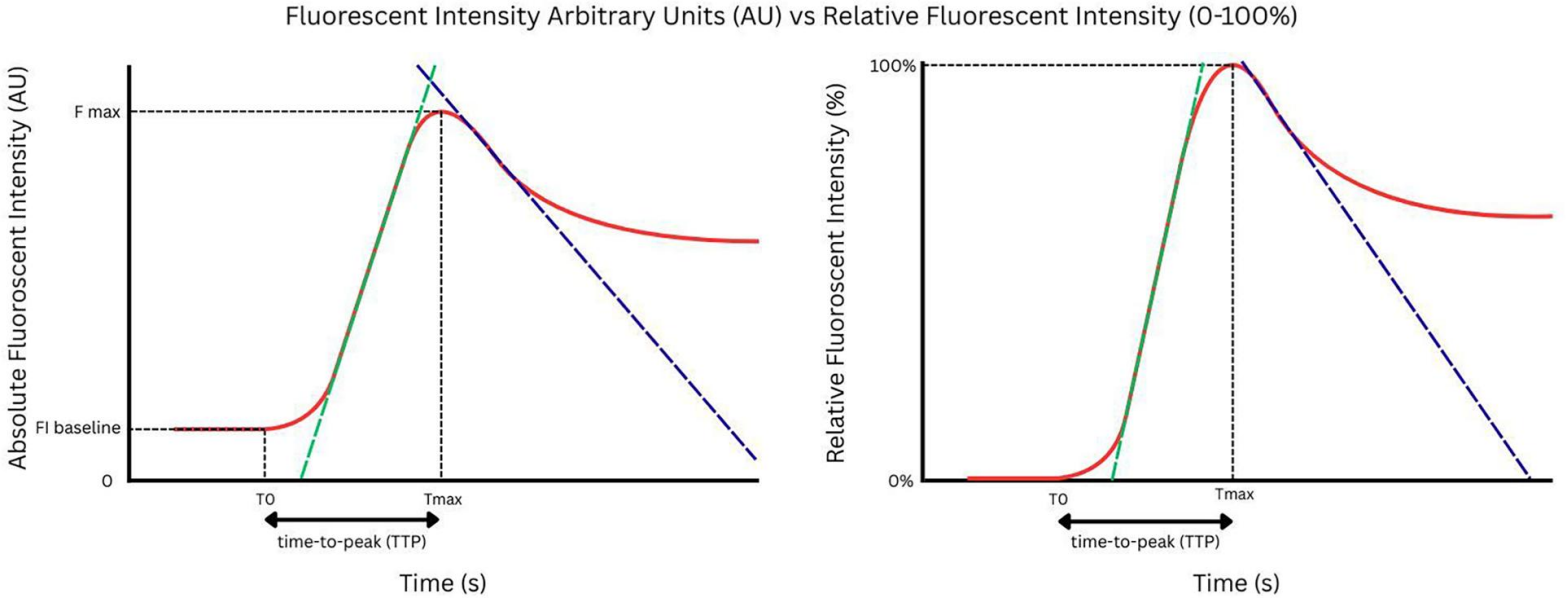
Fluorescence-time curve (FTC) in both absolute (left) and normalized (right) forms. T_0_: The moment when fluorescence intensity within the region ROI first significantly exceeds the background level, measured in seconds (s). F_max_: The maximum fluorescence intensity, measured in arbitrary units (AU). TTP: The time-to-peak, calculated as the difference between T_0_ and the time when F_max_ occurs (T_max_), measured in seconds (s). Green dashed line: inflow rate (AU/s in absolute form, %/s in normalized form). Blue dashed line: outflow rate (AU/s in absolute form, %/s in normalized form). Absolute mean slope inflow: The average rate of fluorescence intensity increase between T_0_ and T_max_, measured in AU/s. Normalized mean slope inflow: The average rate of fluorescence intensity increase in a normalized FTC, expressed as a percentage per second (%/s). Normalized maximum slope inflow: The steepest increase in fluorescence intensity between T_0_ and T_max_ in a normalized FTC, expressed as a percentage per second (%/s). Normalized maximum slope outflow: The steepest decline in fluorescence intensity between T_max_ and the end of the FTC in a normalized curve, expressed as a percentage per second (%/s).

For each recording, both AMS and EPA generated an FTC based on the fluorescence intensity within the predefined ROI. All FTCs were analyzed in both their absolute and normalized forms (see Figure 1**)**. In the absolute form, raw fluorescence intensity values were analyzed directly as recorded by the imaging system and expressed in grayscale arbitrary units (AU). In the normalized form, the baseline fluorescence was set to 0% and maximum intensity (F_max_) to 100%, allowing fluorescence intensity to be expressed as a percentage of F_max_ over time. This approach has been shown to minimize case-specific variability (14). From the absolute and normalized FTCs, the following parameters were calculated:

- **T_0_:** The moment when fluorescence intensity within the ROI first significantly exceeds the background level, measured in seconds (s).
- **F_max_:** The maximum fluorescence intensity, measured in arbitrary units (AU).
- **TTP:** The time-to-peak, calculated as the difference between T_0_ and the time when F_max_ occurs (T_max_), measured in seconds (s).
- **Absolute mean slope inflow:** The average rate of fluorescence intensity increase between T₀ and T_max_, measured in AU/s.
- **Normalized mean slope inflow:** The average rate of fluorescence intensity increase in a normalized FTC, expressed as a percentage per second (%/s).
- **Normalized maximum slope inflow:** The steepest increase in fluorescence intensity between T₀ and T_max_ in a normalized FTC, expressed as a percentage per second (%/s).
- **Normalized maximum slope outflow:** The steepest decline in fluorescence intensity between T_max_ and the end of the FTC in a normalized curve, expressed as a percentage per second (%/s).

The primary outcome was TTP, as previous studies have shown that time-based parameters are less affected by technical factors such as camera settings or acquisition conditions (9). The secondary outcomes were the other parameters: T₀, Fmax, absolute mean slope inflow, normalized mean slope inflow, normalized maximum slope inflow, and normalized maximum slope outflow. Parameter definitions and equations are provided in Supplementary Data Sheet S1.

### 2.4 Statistical analysis

Statistical analysis was performed in Statistical Package for the Social Sciences (SPSS), version 28.0. Descriptive statistics were used to summarize quantitative perfusion parameters. Categorical variables were presented as number of cases (n) and percentages (%). Continuous variables were presented as mean ± standard deviation (SD) or median with range (min–max), depending on the data distribution. The distribution of all continuous variables was assessed visually using histograms and formally tested with the Shapiro–Wilk test. The intraclass correlation coefficient (ICC) was calculated to assess the agreement between platforms for each parameter and interpreted according to Koo and Li (2016) (15). As most parameters were not normally distributed, paired comparisons between AMS and EPA were performed using the Wilcoxon signed-rank test. Bland–Altman plots were generated to visualize systematic differences and limits of agreement (LOA) between the two platforms (16). All statistical tests were two-sided, and a *p*-value <0.05 was considered statistically significant.

## 3 Results

A total of 80 intraoperative ICG-FA recordings from 74 patients were included in the analysis. The cohort consisted of patients undergoing various reconstructive procedures, including fasciocutaneous (*n* = 39), phalloplasty (*n* = 23), muscle (*n* = 11), and osseous (*n* = 2) flaps.

### 3.1 Comparing two software platforms

Excellent agreement was observed for TTP (ICC = 0.979, 95% CI: 0.967–0.987) and normalized mean slope inflow (ICC = 0.944, 95% CI: 0.913–0.964). Good to excellent agreement was found for T_0_, F_max_, and absolute mean slope inflow. By contrast, normalized maximum slope parameters showed poor to moderate agreement, with ICC values of 0.412 for inflow and 0.315 for outflow. A detailed overview of the ICC values for all parameters is shown in Table 1**.**

**Table 1:** Intraclass correlation coefficients (ICC).

Parameter	ICC (95% CI)	Agreement	n
TTP	.979 (.967 -.987)	Excellent	80
T_0_	.895 (.837-.933)	Good to excellent	80
F_max_	.987 (.869–996)	Good to excellent	80
Absolute mean slope inflow	.929 (.883 -.956)	Good to excellent	80
Normalized mean slope inflow	.944 (.913 -.964)	Excellent	80
Normalized max slope inflow	.412 (.088 -.622)	Poor to moderate	80
Normalized max slope outflow	.315 (−0.67 -.569)	Poor to moderate	64^a^

Intraclass Correlation Coefficients (ICC) for the agreement between AMS and EPA for all perfusion parameters. Values represent the degree of consistency between the two platforms, with higher ICC values indicating better agreement.

a Outflow detected in 64/80 ICG-FA recordings.

The Wilcoxon signed-rank test compared the perfusion parameters between AMS and EPA, showing significant systematic differences for six out of seven parameters (Table 2). For TTP, the median AMS value was lower than the EPA value. Nevertheless, AMS produced higher values in most cases, with 68 negative ranks (indicating AMS had higher values) and 12 positive ranks (indicating EPA had higher values), with *p* < 0.001. Normalized mean slope inflow was the only parameter for which no significant difference was found (*p* = 0.158). The ranks were nearly balanced, with 41 positive ranks (indicating EPA had higher values) and 39 negative ranks (indicating AMS had higher values), suggesting no consistent difference between the platforms. The remaining results are provided in Table 2.

**Table 2:** Wilcoxon signed-rank test.

Parameter	AMS	EPA	Positive Ranks (n)	Negative Ranks (n)	p-value
**TTP (s)**	86.8 (10.4–291.8)	93.3 (8.9–279.6)	12	68	<.001
**T_0_ (s)**	36.7 (3.2–177.0)	38.4 (5.1–173.2)	61	19	.001
**F_max_ (AU)**	49.9 (3.2–217.3)	37.1 (2.0–212.1)	13	67	<.001
**Absolute mean Slope inflow (AU/s)**	0.4 (0.0–11.74)	0.4 (0.0–17.0)	53	27	<.001
**Normalized mean slope inflow (%/s)**	1.0 (0.4–8.1)	1.0 (0.2–10.1)	41	39	.158
**Normalized max Slope inflow (%/s)**	3.3 (0.3–15.0)	5.9 (1.8–40.3)	73	7	<.001
**Normalized max slope outflow (%/s)**	0.4 (0.0–5.2)	1.8 (0.1–25.1)	63	1	<.001

Wilcoxon Signed-Rank Test results for parameter comparison between EPA and AMS. Values are presented as median (range). Ranks were calculated as EPA—AMS, where positive ranks indicate EPA > AMS and negative ranks indicate AMS > EPA.

### 3.2 Visualizing agreement

**Figure 2:**
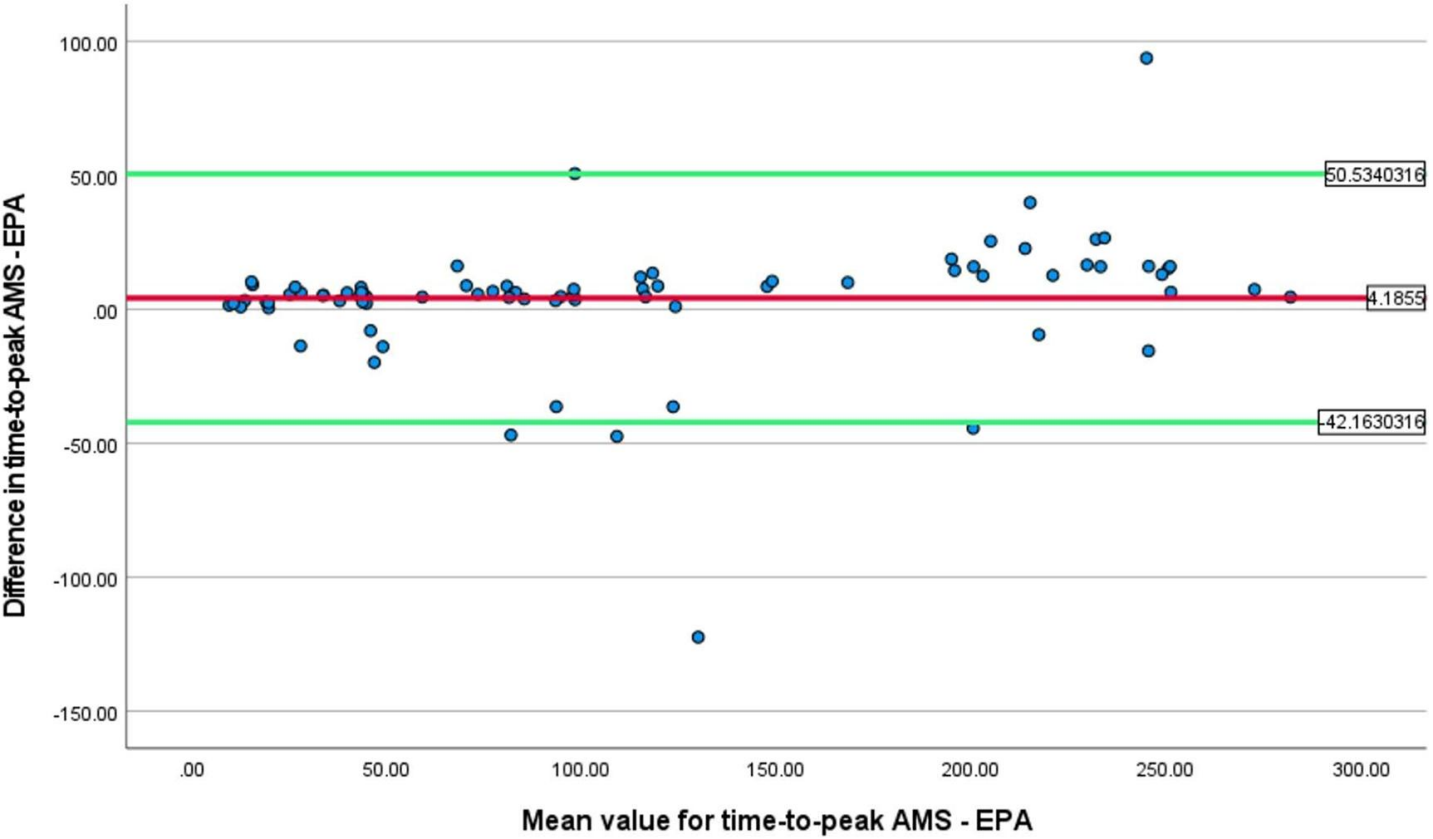
Bland–altman plot for the primary objective: time-to-peak (TTP). The red line represents the mean difference (bias) between the platforms. The two green lines indicate the 95% limits of agreement (LOA), showing the range within which 95% of the differences are expected to fall. Each blue dot represents an individual measurement, and its position indicates the difference between the corresponding TTP values from AMS and EPA.

Figure 2 shows the Bland–Altman plot for TTP values comparing AMS and EPA. The mean difference (bias) was +4.2 s, with AMS showing slightly higher TTP values than EPA on average. The 95% LOA ranged from −42.2 to +50.5 s.

**Figure 3:**
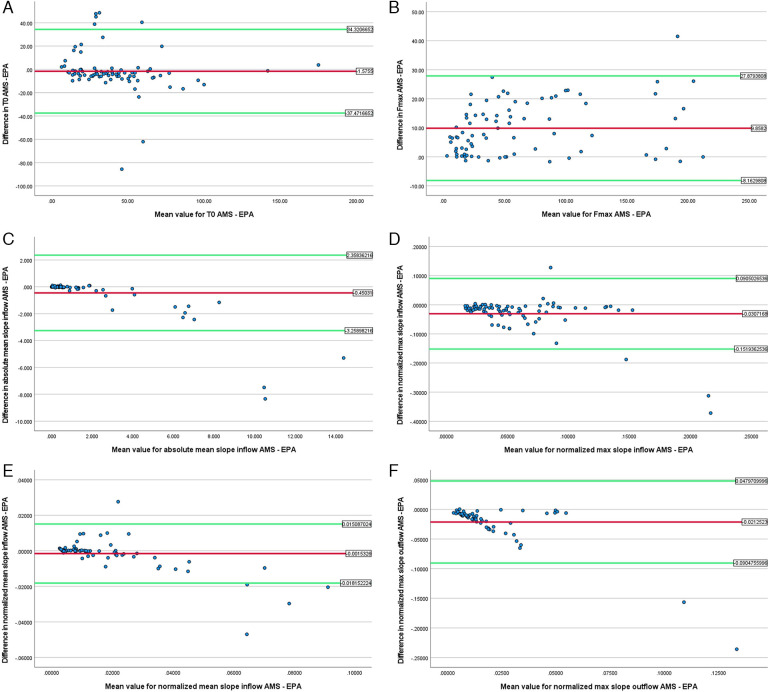
Bland–altman plots for the secondary objectives: T_0_ **(A)**, f_max_ **(B)**, absolute slope inflow **(C)**, normalized mean slope inflow **(D)**, normalized maximum slope inflow **(E)**, normalized maximum slope outflow **(F)**

For the secondary outcomes, the agreement between AMS and EPA varied across parameters, as shown in Figure 3. Normalized mean slope inflow had the best agreement, with a minimal bias of −0.153%/s and narrow LOA (–1.82 to +1.51%/s). Absolute mean slope inflow showed a small bias (–0.450 AU/s) with wider LOA (–3.26 to +2.36 AU/s). For T₀, the bias was −1.58 s, with broad LOA (–37.5 to +34.3 s). Fmax had a bias of +9.80 AU, with LOA ranging from −8.04 to +27.6 AU. Both normalized maximum slope parameters (inflow and outflow) showed poor agreement. For inflow, the bias was −3.07%/s with LOA ranging from −15.2 to +9.05%/s. For outflow, the bias was −2.13%/s, with LOA ranging from −9.05 to +4.80%/s.

## 4 Discussion

Reliable and reproducible quantification is an essential feature of ICG-FA to facilitate perfusion assessment. The present study aimed to evaluate the agreement of Q-ICG-FA parameters between two independent platforms using identical intraoperative recordings. TTP and normalized mean slope inflow were identified as the most reliable parameters, demonstrating excellent reproducibility across platforms. In contrast, parameters derived from maximum slope calculations showed greater variability. These findings highlight the importance of selecting parameters that ensure accurate and consistent perfusion assessments, which is crucial for standardization in both clinical and research contexts.

Previous studies have demonstrated that several Q-ICG-FA parameters are highly sensitive to both technical and patient-related factors such as camera calibration, acquisition settings, injection technique, and tissue characteristics (8, 9, 17). In particular, absolute intensity–based parameters, including F_max_ and absolute slope measures, are significantly affected by variations in illumination, working distance, and camera settings (17). These factors limit the comparability of such parameters across systems and surgical settings. In contrast, time-dependent parameters, such as TTP and normalized parameters that account for differences in fluorescence amplitude, are less susceptible to these variables and tend to produce more consistent results (8). Further supporting this, previous studies demonstrated that normalization of FTCs enhances the repeatability of measurements, thereby increasing the reliability of perfusion quantification (18). The present study successfully identified parameters that are more robust and less susceptible to software-specific processing. However, despite using identical intraoperative recordings, significant differences were still observed in the absolute values of most parameters, with several measurements showing outliers. This suggests that while certain parameters are overall more reliable, variability introduced by processing algorithms remains a significant factor, as demonstrated by Nijssen et al. (14). These findings emphasize that Q-ICG-FA results are not solely influenced by acquisition conditions or patient factors but are also shaped by software-specific processing. Addressing this source of variability is essential to achieve truly reliable and reproducible quantitative perfusion assessments. This understanding is also essential for facilitating the use of thresholds generated with different software platforms.

The degree of reproducibility across software platforms varied considerably between individual perfusion parameters. Because both platforms analyzed the same ICG-FA recordings and identical ROIs, the observed inter-platform differences cannot be attributed to true differences in tissue perfusion within a given recording. Instead, they most likely reflect methodological variability introduced by software-specific signal processing and parameter calculation. Differences in smoothing algorithms may affect the FTCs in different ways. Parameters based on the global curve shape, such as TTP and normalized mean slope inflow, are therefore expected to be relatively robust. In contrast, maximum slope parameters depend on the steepest local segment of the curve and are more sensitive to filtering and residual noise. This parameter-specific sensitivity was also reflected in our results. TTP showed generally high consistency between platforms. However, significant variability was observed in some measurements, with differences exceeding 50 s between the platforms. Review of these outliers showed that the discrepancies were primarily due to differences in the definition and operational detection of T_0_ across the systems. Since TTP is calculated as the time interval between T_0_ and T_max_, differences in T_0_ calculation can have a substantial effect on the calculated TTP values. F_max_ also demonstrated good to excellent agreement, but the wide limits of agreement and systematic bias observed between platforms indicate substantial variability. Upon reviewing the F_max_ results, we found that the values for F_max_ were primarily influenced by differences in curve filtering. Both absolute and normalized mean slope inflow parameters also demonstrated high reproducibility. The normalized mean slope inflow demonstrated excellent agreement between the platforms, with minimal systematic differences and few outliers. This indicates that normalization effectively reduces inter-platform variation by compensating for amplitude-related differences in signal intensity. In contrast, parameters derived from maximum slope calculations demonstrated poor agreement between platforms. These parameters depend on the steepest segment of the FTC, making them highly susceptible to differences in curve smoothing and to small fluctuations in signal noise.

Based on previous research and the present findings, both TTP and normalized mean slope inflow appear to be less sensitive to acquisition-related variability and software-specific processing. As such, they are promising candidates for defining quantitative perfusion thresholds. Among these, normalized mean slope inflow, in particular, demonstrates the least variability and may therefore be the most reliable parameter for establishing such thresholds. Nevertheless, the observed inter-platform differences indicate that even relatively reproducible parameters may not yield directly interchangeable threshold values across software platforms. Therefore, standardized parameter definitions, signal-processing methods, and calculation procedures are essential before Q-ICG-FA thresholds can be reliably implemented across different systems. Moreover, technical reproducibility alone does not guarantee clinical validity. The extent to which these parameters correlate with clinical outcomes remains a critical step toward defining reliable and clinically relevant thresholds.

Most perfusion thresholds currently reported in the literature originate from studies on mastectomy skin flaps, where perfusion failure is predominantly ischemic (19). These thresholds are typically expressed as relative perfusion values, comparing poorly perfused areas to well-perfused reference regions within the same flap (20). In such random-pattern flaps, ischemia often arises from thin skin or excessive tension, and reduced arterial inflow is the main mechanism of failure. In contrast, free flaps and pedicled flaps based on skeletonized perforators or flap vessels are prone to different perfusion challenges. In these reconstructions, complications more commonly involve venous congestion or delayed inflow rather than pure ischemia (21). Consequently, perfusion thresholds established in mastectomy flaps may not be directly applicable to other flap types. Parameters that describe the timing and rate of perfusion, such as TTP and inflow slopes, may provide more clinically relevant information in this context (22). It is essential to identify which parameters are most predictive of perfusion outcomes for different flap types, ensuring their clinical relevance and applicability.

This study has several limitations that should be considered when interpreting the findings. The primary limitation is that only two analysis platforms (AMS and EPA) were compared, one of which is still under development. Therefore, the level of agreement observed here may not be directly applicable to other software systems and should be interpreted as specific to the software versions and processing settings used in this study. Moreover, although identical video recordings were analyzed across platforms, substantial variation in fluorescence intensity and perfusion characteristics existed between recordings. These differences may have influenced how each software processed the fluorescence signal, particularly for absolute intensity–based parameters. Given the non-normal distribution of the data, the results should be interpreted with caution, as the statistical methods used may not fully capture the underlying variability. Finally, this study focused on methodological reproducibility rather than clinical validation, which limits the interpretation of its clinical relevance. Agreement between platforms may be weaker, and differences may be more pronounced in certain cases,.for example, in cases with perfusion-related complications or across different flap types (e.g., free vs. pedicled) or tissue type (e.g., fasciocutaneous, muscle, or bone flaps). However, subgroup analyses by flap type were not performed, as the primary aim was to assess inter-platform agreement using paired measurements of identical recordings, rather than to compare perfusion characteristics between flap types. Moreover, the number of cases within specific subgroups, including individual flap types and free vs. pedicled flaps, was too small to draw reliable conclusions.

Future research should focus on determining which Q-ICG-FA parameters best predict postoperative outcomes. While time-based measures such as TTP appear most promising, their predictive value must be validated in larger outcome-driven studies. Once clinically relevant parameters have been identified, threshold values should be defined and validated across software platforms. To ensure reproducibility and comparability, future studies should clearly report and, where possible, standardize the camera system, analysis software, parameter definitions, signal-processing methods, and analysis settings.

## 5 Conclusion

In conclusion, while Q-ICG-FA shows potential for intraoperative perfusion assessment, the variability introduced by differences in imaging protocols, camera systems, and analysis methods must be addressed to ensure its reliability and reproducibility across clinical settings. The present study suggests that TTP and normalized mean slope inflow are promising candidates for establishing quantitative perfusion thresholds in reconstructive surgery. However, clinical validation of these parameters in larger outcome-driven studies is essential to confirm their predictive value. Furthermore, ensuring methodological transparency and standardization of imaging and analysis protocols will be critical for achieving consistent and reliable perfusion assessments.

## Data Availability

The raw data supporting the conclusions of this article will be made available by the authors, without undue reservation.
