# Supplementary material for: Cross-software comparison shows strong agreement for quantitative indocyanine green fluorescence angiography in reconstructive surgery
Source: Front Surg. 2026 Jun 24;13:1776745. doi: 10.3389/fsurg.2026.1776745 (PMC13341695; doi:10.3389/fsurg.2026.1776745)
Supplement: Supplementary file 1 [file Datasheet1.pdf]

# *Supplementary Material*

## Supplementary Data Sheet S1

## EPA

- **T0** (defined as the last point before the signal reaches ≥10% of its maximum value)

$$T_0 = \max\{t \,|\, F(t) < 0.10 \times F_{\max}\}$$

- **ttp** (Tmax – T0)

$$\mathrm{TTP} = t_{\mathrm{Fmax}} - t_0$$

  - with Tmax defined as the time point of Fmax
- **Fmax** (defined as the highest signal intensity).

$$F_{\max} = \max(fit)$$

  - with Tmax defined as the time point of Fmax
- **Absolute average ingress slope** (defined as the average of the derivative of signal between T0 and Tmax)

$$\text{Absolute Mean Slope}_{\text{in}} = \frac{F_{\max} - F(T_0)}{T_{\max} - T_0}$$

  - Where F(t) is the fluorescence intensity at time t
- **Normalized average ingress slope**

$$\text{Normalized Mean Slope}_{\text{in}} = \frac{100\% - 0\%}{T_{\max} - T_0} = \frac{100}{T_{\max} - T_0}$$

- **Normalized Max ingress slope** (defined as the maximum of derivative of signal between T0 and Tmax)

$$\text{Normalized Max Slope}_{\text{in}} = \max\left(\frac{F(t_{i+1}) - F(t_i)}{t_{i+1} - t_i}\right)$$

  - For $t_i$ is between T0 and Tmax and F(t) is normalized intensity
- **Normalized Min egress slope** (defined as the maximum of the derivative of signal between Tmax and Tend)

$$\text{Normalized Max Slope}_{\text{out}} = \min\left(\frac{F(t_{i+1}) - F(t_i)}{t_{i+1} - t_i}\right)$$

  - For $t_i$ is between Tmax and Tend

**AMS**

- **T0** (defined as the first time point when intensity exceeds a dynamic threshold)

$$\text{Threshold} = \mu_{\text{baseline}} + \max\left(\frac{F_{\text{max}}}{80}, 0.5, 3 \cdot \sigma_{\text{baseline}}\right)$$

    - Where µbaseline, σbaseline: mean and standard deviation of the first second of data.
    - Fmax: maximum fluorescence intensity of the smoothed curve.
- **ttp** (defined as Tmax – T0)

$$\text{TTP} = t_{\text{Fmax}} - t_0$$

    - with Tmax defined as the time point of Fmax
- **Fmax** (defined as the maximum fluorescence intensity of the smoothed curve)

$$F_{\text{max}} = \max(fit)$$

- **Absolute mean slope inflow**
    - Where Z is the baseline fluorescence (mean before T0).

$$\text{Mean Slope}_{\text{in}} = \frac{F_{\text{max}} - Z}{\text{TTP}}$$

- **Normalized mean slope inflow**

$$\text{Norm Mean Slope}_{\text{in}} = \frac{\text{Mean Slope}_{\text{in}}}{F_{\text{max}} - Z}$$

- **Normalized max slope inflow** (defined as the maximum of derivative of signal between T0 and Tmax)

$$\text{Max Slope}_{\text{in}} = \max\left(\frac{d(fit)}{dt}\right) \text{ between } T_0 \text{ and } t_{\text{Fmax}}$$

$$\text{Norm Max Slope}_{\text{in}} = \frac{\text{Max Slope}_{\text{in}}}{F_{\text{max}} - Z}$$

- **Normalized max slope outflow** (defined as the maximum of derivative of signal after Tmax)

$$\text{Max Slope}_{\text{out}} = \max\left(-\frac{d(fit)}{dt}\right) \text{ after } t_{\text{Fmax}}$$

$$\text{Norm Max Slope}_{\text{out}} = \frac{\text{Max Slope}_{\text{out}}}{F_{\text{max}} - Z}$$
